# Chronic myeloid leukemia: incorporation of recent advances into current treatment algorithms

**DOI:** 10.1038/s41408-026-01527-6

**Published:** 2026-05-28

**Authors:** Fadi G. Haddad, Hagop Kantarjian

**Affiliations:** Department of Leukemia, The University of Texas MD Anderson Cancer Center, Houston, TX USA

**Keywords:** Targeted therapies, Myeloproliferative disease

## Abstract

Several approved and investigational BCR::ABL1 tyrosine kinase inhibitors (TKIs) and STAMP inhibitors are used for the treatment of chronic myeloid leukemia in chronic phase (CML-CP). In the frontline setting, multiple factors affect selection of therapy including the goal of treatment, cost of TKIs, CML risk, and patient’s comorbidities. Achieving a complete cytogenetic response (*BCR::ABL1* transcripts on the International Scale [IS] <1%) with TKI therapy within the first year is associated with normalization of survival, whereas achieving a deeper molecular response (*BCR::ABL1* transcripts < 0.01% [IS]) may allow for treatment discontinuation with the possibility of treatment-free remission. Although standard doses are approved for each TKI, post approval studies have demonstrated that an optimal biologic dose is safer than and as effective as the approved dose. In cases of TKI toxicities, reducing the dose rather than switching TKIs is recommended unless the patient experiences a prohibitive toxicity, in which case the treatment should be changed. In patients experiencing failure of frontline therapy due to resistance or intolerance, multiple second- and third-line options are available, including second-generation TKIs, ponatinib, and asciminib, as well as novel investigational agents, including the ABL1 kinase domain inhibitors olverembatinib and ELVN-001 and the STAMP inhibitors TGRX-678 and TERN-701. In this review, we discuss the recent advances in the treatment of CML-CP and challenge some established management practices.

## Introduction

Several recent reviews in the field of Philadelphia chromosome (Ph)-positive chronic myeloid leukemia (CML) have addressed important evolving concepts in disease management. We elaborate on these briefly before moving to discussion of the recent advances and their incorporation into our CML management.

## Important CML management approaches that may differ from our historical established strategies over the past 2 decades

We summarize the CML response definitions of interest to clinical practice in Table 1. These molecular responses have been used in different studies and expert guidelines/recommendations to address their importance, and to suggest indications to continue or change BCR::ABL1 tyrosine kinase inhibitor (TKI) therapy. To simplify: (1) achieving a complete cytogenetic response (CCyR or MR2; equivalent to *BCR::ABL1* transcripts < 1% on the International Scale [IS]) is associated with normalization of a patient’s survival to that of an age-matched normal population. (2) Achievement of a major molecular response (MMR or MR3; *BCR::ABL1* transcripts < 0.1% [IS]) is associated with improved event-free survival (EFS), with the caveat that events may not be defined objectively (e.g., TKI therapy discontinuation because of physician decision or not meeting some molecular response criteria for optimal response). The MMR response was used in several randomized trials as the primary endpoint for TKI regulatory approval, but it does not translate into improved overall survival (OS) among patients who have a MMR versus those who do not. (3) Deep molecular response (DMR) includes achievement of at least a 4-log reduction of *BCR::ABL1* transcript levels (MR4, MR4.5, or MR5; *BCR::ABL1* transcripts < 0.01% [IS]). Achieving a durable or sustained DMR for 2 years or longer is associated with successful TKI discontinuation without later molecular relapse or treatment-free remission (TFR; essentially, a molecular cure) in 35–45% of patients. Achieving a durable DMR for 5 years or longer is associated with a TFR rate of 80% [1, 2].

**Table 1:** Important TKI response categories in patients with CML.

Response		Definition	Translates into
Early molecular response (EMR)		*BCR::ABL1* ≤ 10% (IS) at 3 and 6 months	EMR at 6 months → Significant improvement in overall survival
Complete cytogenetic response (CCyR; MR2)		*BCR::ABL1* (IS) ≤ 1% or ≥2-log reduction in *BCR::ABL1* transcripts	CCyR at 12 months → Improvement in overall survival Loss of MR2 after 1+ year = CML resistance
Major molecular response (MMR; MR3)		*BCR::ABL1* (IS) ≤ 0.1% or ≥3-log reduction in *BCR::ABL1* transcripts	MMR → Modest improvement in event-free survival (definition of events could be subjective); no overall survival benefit
Deep molecular response (DMR)	MR4	*BCR::ABL1* (IS) ≤ 0.01% or ≥4-log reduction in *BCR::ABL1* transcripts	DMR → Possibility of treatment discontinuation if response is sustained for at least 2–5 years
	MR4.5	*BCR::ABL1* (IS) ≤ 0.0032% or ≥4.5-log reduction in *BCR::ABL1* transcripts	
	MR5	*BCR::ABL1* (IS) ≤ 0.001% or ≥5-log reduction in *BCR::ABL1* transcripts	
Major cytogenetic response (MCyR)		*BCR::ABL1* ≤ 10% (IS)	MCyR at 12 months → Reasonable overall survival at 10 years, particularly relevant in elderly patients

**Fig. 1:**
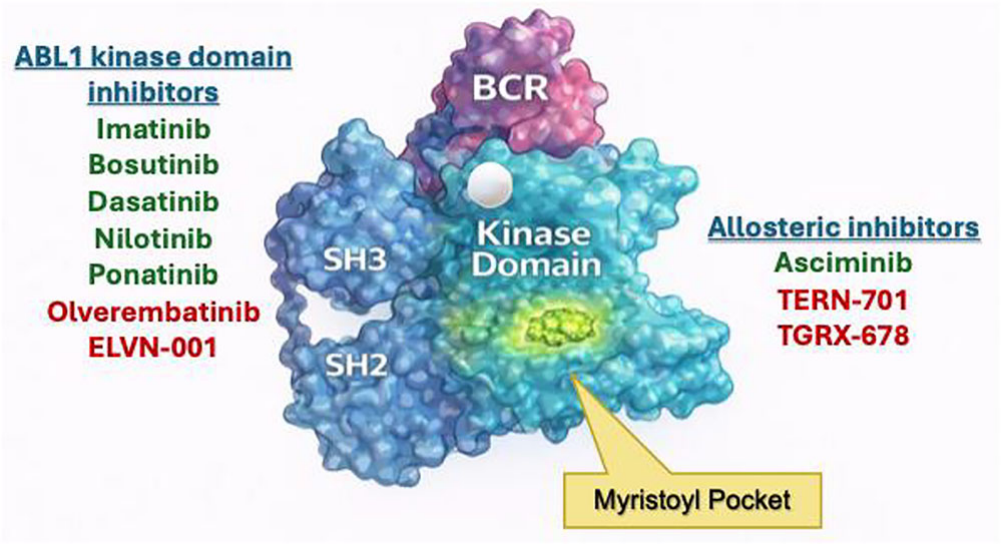
Approved and investigational BCR::ABL1 TKIs and allosteric inhibitors.

Figure 1 summarizes the BCR::ABL1 TKIs approved by regulatory bodies and the investigational ones of interest. Imatinib is referred to as a first-generation TKI because it was the first one developed. It normalizes survival as well as any of the more potent TKIs, is available and affordable to all patients (cost of care <$500/year with generic imatinib in the United States, equivalent to <$15,000 for 30-year or lifetime treatment), and is likely the safest TKI with regard to long-term and prohibitive toxicities. The second-generation TKIs were the next wave of the more potent drugs developed, including dasatinib, bosutinib, and nilotinib. The third-generation TKIs were even more potent and had the unique property of suppressing *T315I*-mutated clones which are resistant to imatinib and the second-generation TKIs. The third-generation TKIs are categorized into two groups based on their binding sites: those inhibiting the ABL1 kinase domain include ponatinib, olverembatinib, and ELVN-001, whereas those that selectively target the ABL myristoyl pocket are called STAMP inhibitors and include asciminib, TERN-701, and TGRX-678.

**Fig. 2:**
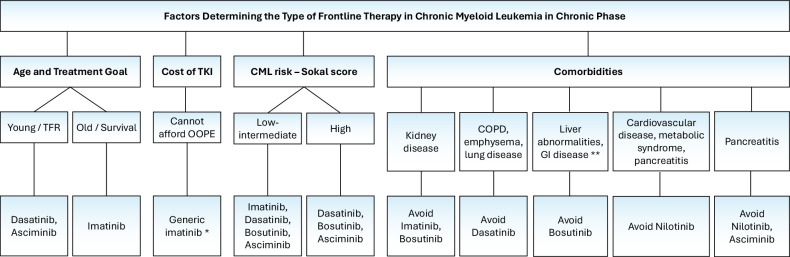
Factors determining the type of frontline therapy for CML-CP at MD Anderson Cancer Center. *Generic imatinib available at less than $500 per year through the Mark Cuban Cost Plus Drug Company. **Includes diarrhea, inflammatory bowel disease, and irritable bowel syndrome. COPD chronic obstructive pulmonary disease, GI gastrointestinal, OOPE out-of-pocket expenses.

Our choices of frontline BCR::ABL1 TKI therapy for CML in chronic phase (CML-CP) and the reasons for such choices are summarized in Fig. 2. The primary aim of CML therapy is to normalize OS. Secondary aims include achievement of TFR, reduction of the incidence of short- and long-term adverse events (AEs), and good “treatment value” (cost-benefit ratio). Thus, the TKI choice is based on the aims of therapy (survival versus TFR), existing co-morbidities, CML risk, and cost of care. When OS is the primary endpoint, generic imatinib fulfills the aims of therapy: it normalizes OS, is cost-effective, and is safe. When achieving an earlier DMR/TFR is important (e.g., for younger patients) or in high-risk CML cases, generic dasatinib administered at 50 mg daily is an excellent choice. The choice of TKI may also vary based on the patient’s existing co-morbidities, the cost of the drug, and the affordability or availability of the drug in a particular geography.

Some established CML management traditions, principles, and/or recommendations may not be supported by the long-term updates of clinical trials and other landmark CML studies. These are summarized in Table 2 and have been detailed in several recent reviews [3–8].

**Table 2:** Revisiting established practices in the management of CML-CP.

Established Practices	Considerations
Use TKIs at MTD	Use TKIs at OBD, a lower dose, safer and as effective as the higher dose (e.g., dasatinib 50 mg/day in frontline, ponatinib 15–30 mg/day in later lines)
Ignore the cost of therapy when choosing the TKI	Consider the “treatment value” (cost:benefit) of the TKIs (e.g., generics of imatinib when survival is the treatment goal)
Define “failure” of frontline TKI therapy as both intolerance and resistance (e.g., high rates of approximately 40%)	Restrict the term “failure” to true disease resistance (*BCR::ABL1* > 1% [IS]; approximately 10% at 10 years) rather than intolerance
Switch TKIs if toxicity	Reduce the TKI dose if toxicity rather than switch If toxicity recurs or if prohibitive (e.g., pulmonary hypertension, arterio- or veno-occlusive disease, pancreatitis, other), then switch
Change TKI in patients failing to achieve *BCR::ABL1* ≤ 0.1% (IS)	Maintain the same TKI and dose in patients in CCyR even if a MMR is not achieved
Pursue TFR by rotating TKIs for *BCR::ABL1* > 0.01% (IS) in patients who are otherwise in CCyR or MMR	Maintain the same TKI and dose in patients in CCyR or MMR Rotate TKIs with the goal of achieving a DMR might lead to more adverse events while not achieving the desired goal
Switch TKIs in all patients with CML who do not achieve *BCR::ABL1* ≤ 10% [IS] after 12 months of therapy	TKI treatment could be maintained in older patients with CML not achieving a *BCR::ABL1* ≤ 10% (IS) after 12 months of therapy as they may still have a normal life in chronic phase

*CCyR* complete cytogenetic response (*BCR::ABL1* ≤ 1% [IS]), *CML* chronic myeloid leukemia, *DMR* deep molecular response (*BCR::ABL1* ≤ 0.01% [IS]), *IS* International Scale, *MMR* major molecular response (*BCR::ABL1* ≤ 0.1% [IS]), *MTD* maximum tolerated dose, *OBD* optimal biologic dose, *TKI* tyrosine kinase inhibitor.

**Fig. 3:**
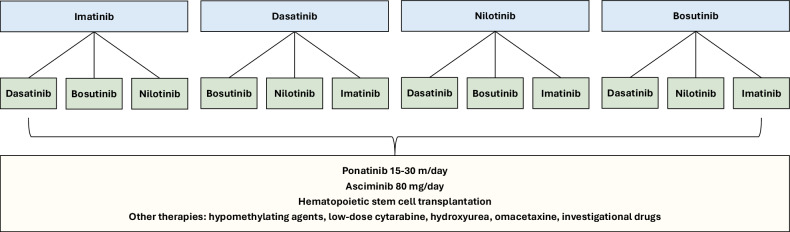
Management of non-prohibitive TKI toxicities. The TKI dose should always be adjusted before considering a change of therapy. When changing to a new TKI, doses lower than the full approved dose can be used if the patient is having a good molecular response. If a new TKI produces toxic effects, second-generation TKIs can be rotated before moving to a third-generation TKI. Although experience with lower doses of asciminib is limited, our experience is that asciminib doses of 20–40 mg daily can be used and are effective. Of note is the potential for TKI cross-intolerance: patients with manifestations of intolerance to one TKI may be more prone to experiencing the same AE or different AEs with other TKIs. When deciding on the second- or third-line therapy, the TKI should be selected according to the patient’s comorbidities.

**Fig. 4:**
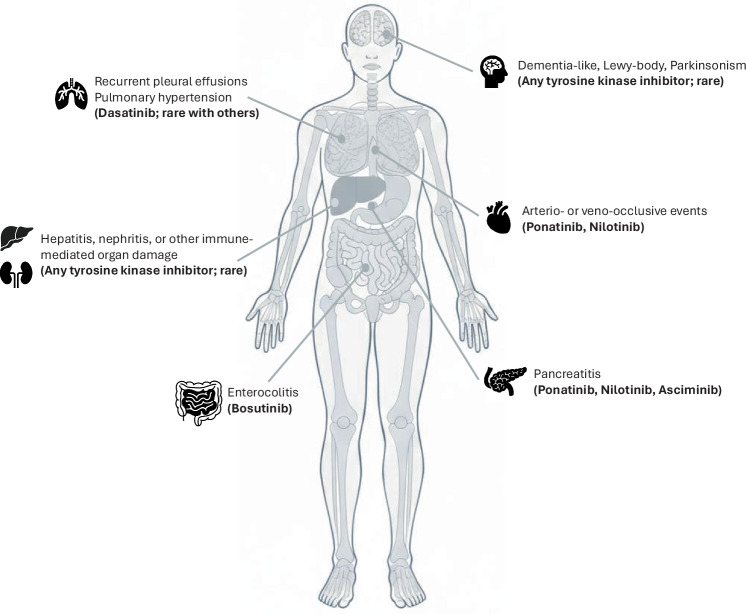
Prohibitive TKI toxicities requiring a change of therapy rather than a dose reduction.

Historically, the development of a TKI toxicity often resulted in a change in the TKI, based on the belief that reducing the TKI dose may result in loss of molecular response. We now know that this is not the case and that patients who have a good molecular response (CCyR, MMR, or a deeper response) but who experience AEs can continue receiving the same TKI at a lower dose. The management of TKI toxicities is depicted in Figs. 3 and 4. Essentially, a dose reduction rather than a change in TKI is recommended in cases of non-prohibitive toxicities, whereas switching to a different TKI is warranted in cases of prohibitive toxicities.

The use of a TKI at the optimal biologic dose (OBD) versus the U.S. Food and Drug Administration (FDA)–approved dose (historically defined as the maximum tolerated dose [MTD]) has also been discussed in recent reviews and is summarized in Table 3 [3–8].

**Table 3:** Variations in the dosing schedules for approved BCR::ABL1 TKIs.

Tyrosine kinase inhibitor	Maximum tolerated dose (FDA approved), mg/day		Optimal biologic dose, mg/day	Lowest effective dose, mg/day
	Frontline	Later line		
Imatinib	400	400	400	100–300
Dasatinib	100	100	50	20
Nilotinib	300 BID	400 BID	300 BID	150–200
Bosutinib	100–400	500	400	100–300
Ponatinib	N/A	45	30 if non-T315I; 45 if T315I	10–15 (once *BCR::ABL1* transcript < 1%)

*BID* twice daily, *FDA* U.S. Food and Drug Administration, *N/A* not applicable.

**Fig. 5:**
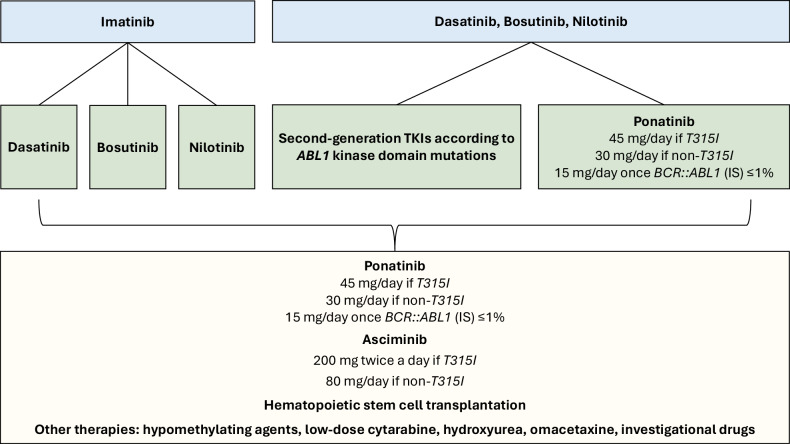
Management of CML resistance to TKI therapy. *ABL1* kinase domain mutational analysis should always be performed to direct TKI selection. Second-generation TKIs should not be rotated if resistance develops unless a guiding mutation is present. Allogeneic stem cell transplantation should be considered for any patient with resistance to a second-generation TKI as it is a one-time, cost-effective, and highly curative procedure (albeit associated with morbidity and mortality [5–10%]).

The management of CML resistance to TKIs is shown in Fig. 5. We use a simple definition of TKI resistance at MD Anderson Cancer Center: *BCR::ABL1* transcripts > 1% (IS) after 1 year of TKI therapy. This contrasts with more elaborate definitions described by established CML groups [3–8].

## Frontline CML therapy: important updates

Today, frontline CML therapy is managed using any of the five approved TKIs: imatinib, dasatinib, bosutinib, nilotinib, and asciminib (the only approved STAMP inhibitor). An important issue often discussed by advisory boards and in symposia is the potential benefit of using asciminib versus imatinib or second-generation TKIs like dasatinib at 50 mg daily or nilotinib. Do any clear-cut benefits justify the cost differentials (≥$300,000/year for asciminib in the United States versus <$500/year for imatinib and $1600/year for dasatinib at 50 mg daily) (Table 4)? A potential justification is a higher 12-month MMR rate with asciminib than with another TKI or imatinib (study endpoints of the ASC4FIRST trial). However, the study endpoints do not correlate with improvement in survival (nor are they expected to) or with increased TFR rates [9, 10]. The ASC4FIRST update at 2.2 years continued to show the improved MMR rate with asciminib than with other TKIs. The 2-year MMR rates for asciminib and investigator-selected TKIs were 74.1% and 52.0%, respectively. The 2-year MMR rates for asciminib and second-generation TKIs were 72.0% and 56.9%, respectively [10].

**Table 4:** Wholesale acquision costs and average wholesale prices for 1 year of TKI therapy in the United States.

Tyrosine kianse inhibitor	Dose (mg/day)	WAC (U.S. dollars)	AWP (U.S. dollars)
Imatinib	400	121,500	145,800
Imatinib generic (MCCPDC)	400	308	414
Dasatinib	100	219,000	262,700
Dasatinib	50	121,500	145,800
Dasatinib	20	60,700	72,900
Dasatinib generic (MCCPDC)	50–100	1300–2600	1600–3100
Nilotinib	300 BID	290,000	347,800
Nilotinib	150 BID	144,900	173,900
Nilotinib generic (MCCPDC)	150–300 BID	7500–15,100	8800–17,500
Bosutinib	400	267,000	320,400
Ponatinib	45, 30, or 15	286,800	344,200
Asciminib	80	268,400	322,100
Asciminib	200 BID	536,800	644,100

*AWP* average wholesale price, *BID* twice daily, *MCCPDC* Mark Cuban Cost Plus Drug Company, *WAC* wholesale acquisition cost.

Of note, ASC4START is a randomized phase 3 trial of asciminib versus nilotinib in Europe in which the study endpoint is treatment discontinuation because of AEs. The study met its primary endpoint, but the early discontinuation rate in the control arm (nilotinib; 5% discontinuation in the first 6 months) was higher than expected given the results of historical trials in which nilotinib was the (preferred) investigational arm. These results highlight perhaps a sub-conscious desire/preference by investigators for the investigational arm (asciminib). Of note, the rate of 12-month MMR (the primary endpoint in the ASC4FIRST trial, the secondary endpoint in the ASC4START trial) for asciminib (57.7%) was similar to that for nilotinib (57.4%) [11].

In an interim analysis, 50 patients with newly diagnosed CML-CP at MD Anderson have received asciminib at 80 mg daily. The cumulative CCyR, MMR, and MR4 rates by 12 months of therapy were 96%, 69%, and 39%, respectively. With a median follow-up time of 12 months, five patients (10%) discontinued therapy (owing to pancreatitis in two patients, acute coronary syndrome in one patient, brain stroke in one patient, and treatment resistance in one patient) [12].

Thus, based on maturing results, there is a need to continue to follow the studies updates on the frontline treatment efficacy and toxicity of asciminib versus other TKIs to identify its treatment value (benefits versus cost).

## Optimizing TKI dose reductions/adjustments

Use of the OBDs of TKIs has moved from post-regulatory approval investigations (e.g., the OPTIC trial of ponatinib at 45-, 30-, and 15-mg starting doses; dasatinib at 50 mg daily) to early phase 1 trials (FDA Project Optimus) that define the MTD (sometimes adjusted to one dose level below the MTD in classical phase 2–3 trials) and a lower dose, presumed to be the OBD based on early efficacy/toxicity data as well as pharmacokinetic and pharmacodynamic data. Defining the OBD, which is presumably as effective as the MTD but much less toxic in the long run, may remain a dynamic process in which researchers continue to explore TKI dose schedules even when a drug becomes available after regulatory approval. The MTDs, OBDs, and lowest effective doses of TKIs used in cases of toxicities are shown in Table 3.

Dose optimization for ponatinib and dasatinib has been described in several reports and reviews [13–15]. In similar articles, early dose-adjustment schedules for bosutinib were reported (100 mg daily × 1–2 weeks, 200 mg daily × 2–4 weeks, 300 mg daily, then 400 mg daily or lower doses based on molecular response and toxicity) [16, 17]. Nilotinib dose-schedule optimization has not been greatly explored [18, 19], although dose reductions to 150 mg twice daily or 200 mg daily are common in patients having a good molecular response, such as an MMR (*BCR::ABL1* transcripts < 0.1% [IS]).

At MD Anderson, 149 patients with newly diagnosed CML-CP (median age, 47 years; low-intermediate Sokal risk score, 95%) received dasatinib at 50 mg daily (*n* = 83) or dasatinib plus venetoclax (*n* = 66). With a median follow-up of 6 years, the cumulative response rates at 5 years were as follows: MMR, 95%; MR4 (*BCR::ABL1* transcripts < 0.01% [IS]), 87%; MR4.5 (*BCR::ABL1* transcripts < 0.0032% [IS]), 86%. The 5-year OS rate was 98%, and the EFS rate was 96%. No cases of transformation to accelerated or blast phase have been observed. Three patients died of causes unrelated to CML. Seventeen patients changed TKI therapy because of suboptimal response or resistance (5%) or serious AEs (5%), for an annual TKI discontinuation rate of only 2% [13].

A recent randomized study in India compared dasatinib administered at 70 mg versus 100 mg daily in 120 patients with CML-CP (median age, 39 years [range, 29–50 years]; 32% high-risk). It demonstrated similar efficacy at 70 and 100 mg daily, with 12-month MMR rates of 81% and 71%, respectively, and 12-month MR4 rates of 28% and 27%, respectively. Toxicities like myelosuppression and pleural effusions were less common with 70 mg of dasatinib than with 100 mg: thrombocytopenia, 15% versus 43% (*P* = 0.029); pleural effusions, 5% versus 10% [20].

The current practice is to reduce the TKI dose rather than switch TKIs in situations of non-prohibitive toxicities, but to switch TKIs if prohibitive toxicities are noted. A prohibitive toxicity is one that may cause irreversible organ damage (e.g., recurrent pleural effusions, pulmonary hypertension, arterio/vaso-occlusive events), organ inflammation (e.g., immune-mediated pneumonitis, pericarditis, myocarditis, hepatitis, nephritis, enterocolitis), or unusual neurotoxicity.

One question posed in current practice that has not been well investigated is when to proactively (preventively) reduce a TKI dose in a patient on a full TKI dose schedule who is tolerating the treatment well without significant toxicities. Several authors have reported on TKI dose reductions prior to attempting a TFR and that these strategies did not reduce TKI efficacy [19, 21, 22]. In addition, investigators in the Czech Republic reported on 207 patients with CML treated with TKIs (imatinib, 79%; median duration of TKI use, 8.6 years) who had the TKI dose reduced by 50% for 6 months and then further reduced to every other day for 6 months before TKI discontinuation. Their 15-month TFR rate was 70% [23]. Iurlo and colleagues reported on 107 patients with CML-CP treated with nilotinib at 300 mg twice daily for at least 3 years and who had a DMR for at least 1 year for whom nilotinib administration was reduced to 300 mg daily for 1 year and then discontinued (92% reduced; 84% discontinued). The TFR rate at 24 months was 71% [24]. Furthermore, Li and colleagues reported on 837 patients with CML-CP or CML in accelerated phase (CML-AP) who had an MMR while receiving TKIs; 271 patients received a reduced TKI dose after a median MMR time of 31 months. The cumulative MMR rate at 6 years was 66% for the full TKI dose versus 75% for the lower TKI dose. The 6-year progression-free survival and OS rates were 99% and 100%, respectively [25].

In our practice, we proactively reduce the TKI dose for a non-symptomatic or mildly symptomatic patient (e.g., with grade 1 fatigue) for the following reasons: (1) MMR or DMR (MR4) for several years to avoid new AEs or improve quality of life; (2) cost-containment issues for a patient with an MMR or DMR (out-of-pocket expenses); (3) patient reluctance to attempt TKI discontinuation when indicated (e.g., DMR for at least 5 years, TFR attempt); (4) patients receiving nilotinib at 300–400 mg twice daily and having a good molecular response (MMR or better) to reduce the potential risk of long-term arterio/vaso-occlusive events or accelerated arteriosclerosis; (5) patients receiving ponatinib at 30–45 mg daily who have experienced MR2 (based on the OPTIC trial results); and (6) patients receiving a TKI known to increase the risk of particular AEs based on existing co-morbidities (e.g., dasatinib-based therapy in a patient with chronic lung problems, bosutinib-based therapy in a patient with colitis or renal dysfunction).

## *ASXL1* variants in CML: what do they mean?

Additional genomic abnormalities (AGAs) encompass both Ph-associated rearrangements (18%) and blood cancer-related gene variants (CGVs) or somatic mutations/variants (SVs). In several studies, CGVs by next-generation sequencing (NGS) were reported in 20–25% of newly diagnosed patients, mostly involving *ASXL1* variants (8–12%). *ASXL1* variants/mutations have been associated with recurrent cytopenias in patients receiving TKI therapy, resulting in frequent treatment interruptions, reduced rates of molecular responses, inferior EFS, and increased rates of mutations in the *ABL1* kinase domain site. However, OS was not worse, perhaps owing to good later-line therapies, or the fact that some form of effective TKI therapy still normalizes early survival even without achievement of good molecular milestones [26, 27].

In an analysis at MD Anderson, targeted NGS was conducted in 115 patients with newly diagnosed CML, including 71 in chronic phase, 10 of whom (14%) had *ASXL1* mutations [28]. *ASXL1* mutations were associated with worse EFS (median, 32.8 months versus 88.3 months; *P* = 0.002), an effect confirmed by multivariate analysis (hazard ratio, 4.25; *P* = 0.004). No difference in OS was noted [28].

In another study, 61 of 200 patients analyzed (31%) had AGAs (including CGVs and Ph-associated variants), with *ASXL1* mutations being the most frequent (18 patients [9%]). *ASXL1* mutations were associated with worse failure-free survival (FFS) than were other AGAs and no AGAS, with 4-year FFS rates of 60%, 75%, and 81%, respectively (*P* = 0.045). Among patients receiving TKI therapy, *ASXL1* mutations were associated with a higher rate of *ABL1* kinase domain mutations than were other AGAs and no AGAs: 20%, 12%, and 2%, respectively (*P* < 0.001) [29].

The same investigators also analyzed 315 patients in four TKI trials to assess whether the more potent TKIs would overcome the negative effect of *ASXL1* mutations. They reported AGAs in 34% of the patients (Ph rearrangements in 18% and SVs in 20%), including *ASXL1* mutations in 7%. TKI therapy was imatinib in 120, nilotinib in 80, dasatinib in 80 and asciminib in 100 patients. The negative impact of Ph-rearrangement with imatinib was overcome with more potent TKIs. *ASXL1* variants/mutations were associated with a worse 12-month MMR rate (55% versus 83%; *P* = 0.001) and a worse 2-year FFS rate (61% versus 91%; *P* < 0.001). *ASXL1* variants/mutations were also associated with increased rates of developing *BCR::ABL1* kinase domain mutations during TKI therapy. Of note, these rates were higher for asciminib than for nilotinib and dasatinib, with a 2-year rate with nilotinib or dasatinib of 21.0% versus 0.6% (*P* = 0.001) and a 2-year rate with asciminib of 56% versus 3–6% (*P* < 0.001). In a multivariate analysis, both CGVs and *ASXL1* mutations were predictors of these outcomes. The inferior outcomes with CGVs (MMR and FFS) were similar with imatinib or more potent TKIs. The development of *BCR::ABL1* kinase domain mutations was associated exclusively with the presence of *ASXL1* mutations at diagnosis [30].

In the HARMONY Platform, among 468 patients with newly diagnosed CML-CP treated with imatinib, 20.5% had SVs, including *ASXL1* mutations in 11.3% (*TET2*, *DNMT3A*, and *RUNX1* mutations in 2–3% each). Somatic variants were associated with lower 5-year rates of progression-free survival (76% vs. 83%; *P* = 0.04) and FFS (44% vs. 65%; *P* < 0.0001) and with a higher 5-year rate of *BCR::ABL1* mutations (20% vs. 11.4%; *P* = 0.067). The researchers observed no differences in survival according to the presence or absence of SVs, however. Also, *ASXL1* mutations were not associated with differences in OS or progression-free survival. They did note differences in FFS according to SVs and *ASXL1* mutations [31].

In summary, patients with SVs/CGVs, particularly those with *ASXL1* variants/mutations, should be monitored closely for responses to and outcomes of TKI therapy and for the development of *BCR::ABL1* kinase domain mutations. TKI therapy may be associated with recurrent cytopenias requiring frequent TKI therapy interruptions. Determining whether the worse outcomes are related to intrinsically worse disease or to TKI interruptions requires further investigation. Single-cell analysis studies may better define whether these SVs occur in the same Ph-positive cells or in separate Ph-negative stem cells (and persist, causing recurrent cytopenias).

## *ABL1* kinase domain mutational testing

Testing for *ABL1* kinase domain mutations is not recommended in patients with newly diagnosed untreated CML, as these mutations are not detected in this setting. Testing for *ABL1* kinase domain mutations is recommended in all patients who develop resistance to TKI therapy. The European LeukemiaNet (ELN) recommends testing for *ABL1* kinase domain mutation in patients who fail to achieve a *BCR::ABL1* transcript ≤ 10% (IS) at 3 months, or ≤1% (IS) at 6 months, or ≤0.1% (IS) at 12 months or any time thereafter [32]. However, many patients might be short of achieving these milestones while others might have a delayed response. Therefore, our approach is to perform *ABL1* kinase domain mutation testing in patients who do not achieve a CCyR (or *BCR::ABL1* transcript ≤1% [IS]) after 12+ months of treatment or lose a CCyR (*BCR::ABL1* transcript increasing to more than 1% [IS]) while on therapy.

The detection of a TKI-resistant mutation should trigger a change of therapy and can guide the selection of the best next TKI based on the sensitivity profile. Over the past decade, molecular technologies for mutation analysis have significantly evolved, with the advent of NGS and digital PCR that are replacing Sanger sequencing as the gold standard *ABL1* kinase domain mutation testing [33–35]. Different studies have shown that NGS has a greater sensitivity which may provide a more accurate result and could better pick up emerging TKI-resistant mutations [33, 36–38].

## Later line TKI therapies and novel investigational TKIs

Today, two third-generation TKIs are available in CML practice: ponatinib, likely the most potent TKI but associated with serious AEs (hypertension, arterio/vaso-occlusive events, pancreatitis, skin rashes; less common at 15–30 mg/day than at 45 mg/day), and asciminib, a STAMP inhibitor that is also very potent but less toxic than ponatinib. Two novel investigational agents, olverembatinib and ELVN-001, inhibit the ABL1 kinase domain like ponatinib. Two novel investigational STAMP inhibitors are TGRX-678 and TERN-701.

### Second-line therapy with ponatinib or asciminib

Second-line therapy with second-generation TKIs post imatinib failure (resistance or intolerance) has been associated with CCyR rates of 50%, MMR rates of 40%, and 7-year OS rates of about 65–75%. Efficacy is similar with dasatinib, bosutinib or nilotinib.

Whereas the experience with third-generation TKIs (ponatinib, asciminib) in third-line CML-CP therapy has been plentiful, less is reported about their efficacy as second-line therapy. Andorsky and colleagues reported interim results from the phase 2 ASC2ESCALATE trial of asciminib given at 80 mg daily as second-line therapy in 101 patients with CML-CP after failure of prior treatment with a TKI. The prior therapy was with dasatinib in 44% of the patients, imatinib in 42%, nilotinib in 9%, and bosutinib in 5%, with 66% of the patients receiving the prior TKI longer than 1 year. The reasons for discontinuing the TKI were lack of efficacy in 56% of the patients and intolerance in 44%. *BCR::ABL1* transcripts were 1–10% (IS) in 31% and greater than 10% (IS) in 30%, suggesting that CML-CP in this population treated was perhaps not highly resistant. On asciminib therapy, the CCyR rate at 6 months was 82%; the 6-month MMR rate was 44%: 58% if prior intolerance and 35% if prior lack of efficacy [39].

Weiming and colleagues reported on 47 patients with CML-CP treated with olverembatinib as second-line therapy; 92% had resistance to frontline TKI therapy; 75% were treated with second-generation TKIs. The 24-month CCyR rate was 90% and MMR rate 58% [40].

### Olverembatinib

In a phase 1/2 study of patients with CML, 165 of them (80% after taking at least two TKIs) received olverembatinib at 40 mg every other day. Among these patients, 123 had CML-CP, and 38 had CML-AP. A *T315I* mutation was present in 77% of the patients. In those with CML-CP, the CCyR rate was 69%, the MMR rate was 56%, the MR4 rate was 44%, and the 3-year OS rate was 94%. In the patients with CML-AP, the CCyR rate was 47%, the MMR rate was 45%, the MR4 rate was 39%, and the 3-year OS rate was 71% [41].

In a study in China, researchers randomized 144 patients post resistance or intolerance to 3+ TKIs (randomization, 2:1) to treatment with olverembatinib (*n* = 96) or best available therapy (*n* = 48) after development of resistance to or intolerance of three or more TKIs. The primary study endpoint, EFS rate at 2 years, was 47% for olverembatinib versus 17% for best available therapy (*P* < 0.001) [42].

Another study in China demonstrated that among 80 patients treated with olverembatinib at 30, 40, or 50 mg every other day, 78% previously received three or more TKIs, 57% had prior exposure to ponatinib, and 31% had prior exposure to asciminib. In CML-CP cases, the CCyR rate was 61%, and the MMR rate was 42%. Among patients with disease resistant to ponatinib, the CCyR rate was 53%, and the MMR rate was 43%. Finally, among patients with prior asciminib exposure, the CCyR rate was 50%, and the MMR rate was 33% [43].

### ELVN-001

Researchers studied 90 patients with CML-CP treated with ELVN-001 at doses ranging from 10 to 80 mg twice daily. The *BCR::ABL1* transcripts at baseline were greater than 1% (IS) in 52% of them. Prior TKI therapy was discontinued for lack of efficacy in 72% of the patients. The median number of prior TKIs was three (range, 1–7); 58% were previously exposed to asciminib, and 43% were previously exposed to ponatinib. The CCyR rate by 6 months was achieved in 52%, and the MMR rate in 32% [44].

### TERN-701

In a study with 63 patients having CML-CP treated with TERN-701 at doses ranging from 160 to 500 mg daily, baseline *BCR::ABL1* transcripts were greater than 10% (IS) in 44% of them. Prior TKI therapy was discontinued for lack of efficacy (according to ELN criteria) in 64% of the patients. The median number of prior TKIs was three. Among 38 patients evaluable for MMR, the MMR rate was 64%. Also, 29% of the patients experienced MR4 or deeper responses [45].

### TGRX-678

In another study, TGRX-678 was given to 158 patients with CML: 108 having CML-CP, and 50 having CML-AP. The dose schedule was 10–80 mg administered twice daily or 40–240 mg administered daily. The median time from diagnosis to treatment was 93 months. Three or more TKIs were received by 66% of the patients with CML-CP and 88% of those with CML-AP. The baseline *BCR::ABL1* transcripts were greater than 10% (IS) in 84% of the patients with CML-CP and 92% of the patients with CML-AP, attesting to the highly resistant nature of the disease in these patients. In the CML-CP cases, the 18-month cumulative CCyR rate was 40%, and the 18-month cumulative MMR rate was 26%. Among patients with CML-CP and a *T315I* mutation, the 18-month CCyR rate was 69%, and the 18-month MMR rate was 50%. In those with CML-AP and a *T315I* mutation, the CCyR rate was 30%, and the MMR rate was 15% [46]. In an update of this study, 53 patients with *T315I*-mutated CML (32 with CML-CP, 21 with CML-AP) treated with TGRX-678 were analyzed. In those with CML-CP, the 2-year cumulative CCyR rate was 59%, the 2-year cumulative MMR rate was 45%, and the 2-year OS rate was 91%. In the patients with CML-AP, the 2-year cumulative CCyR and MMR rates were 29% and 15%, respectively, and the 2-year OS rate was 84% [47].

We questioned whether any of the four novel investigational TKIs are clearly more effective and/or less toxic than the others. From the reported data thus far, all four TKIs appear to be well tolerated, with minimal severe AEs. All four TKIs also appear to be quite effective, but the data is blurred by the different criteria to define resistance (or lack of efficacy) and by the reporting of responses as cumulative (by) or at particular time points (at). Perhaps a reasonable evaluation would be to compare the efficacy rates for these four TKIs among patients who had true resistance to prior treatment with ponatinib or asciminib. Our assessment thus far is that all four TKIs have produced encouraging results regarding their efficacy and toxicity and will hopefully have a timely path toward regulatory approval.
